# Supplementary material for: Chromothripsis during telomere crisis is independent of NHEJ, and consistent with a replicative origin
Source: Genome Res. 2019 May;29(5):737–49. doi: 10.1101/gr.240705.118 (PMC6499312; doi:10.1101/gr.240705.118)
Supplement: Supplemental Material [file supp_gr.240705.118_Supplemental_file_1.zip › contigs/annotated_contigs/DB53/contig.2.DB53_length_937_mean_cov_7.57310565635.docx]

**DB53_length_937_mean_cov_7.57310565635**

```
CAGAGACTAGGATTGCAACCCCTGCCTTTTTTTGTTTTCCATTTGCTTGGTAGATCTTCCTCCATCCTTTTATTTTGAGCCTATGTGTG
 >chr12:87494095-87494402 - E=1e-163
TCTCTGCCCGTGAGATGGTTTCCTGAATACAGCACACTGATGGGTCTTGACTCTTTATCCAATTTGCCAGTCTGTGTCTTTTAATTGGA

GCATTTAGTGCATTTACACTTAAAGTTAATATTGTTATGTGTGAATCTGATCCTGTCATTGTGATGTTAGCTGGTTATTTTGCTCATTA

GTTGATGCAGTTTCTTCCTAGCCTCGATGGTCTTTAC|ATT|CAACATAGTGTTGGAAGTTCTGGCCAGGGCAATTAGGCAGGAGAAGG
                                      >chr8:84352893-84353526 - E=0e+00
AAATAAAGGGTATTCAATTAGGAAAAGAGGAAGTCAAATTGTCCCTGTTTGCAGACGACATGATTGTATATCTAGAAAACCCCATTGTC

TCAGCCCAGAATCTCCTTAAGCTGATAAGCAACTTCAGCAAAGTCTCAGGATACAAAATCAATGTACAAAAATCACAAGCATTCTTATA

CACCAACAACAGACAAACAGAGAGCCAAATCATGAGTGAACTCTCATTCACAATTGCTTCAAAGAGAATAAAATACTTAGGAAGCCATC

TTACAAGGGACGTGAAGGACCTCTTCAAGGAGAACTACAAACCACTGCTCAGTGAAATAAAAGAGGATACAAAGAAATGGAAGAACATT

CCATGCTCATGGGTAGGAAGAATCAATATCGTGAAAATGGCCATACTGCCCAAGGTAATTTACAGATTCAATGCCATCCCCATCAAGCT

ACCAATGACTTTCTTCACAGAATTGGAAAAAACTACTCTAAAGTTCATATGCAACCAAAAAGAGCCCGCATCGCCAAGTCAATCCTAAG

CCAAAAGAACAAAGCTGGAGCCATCACACTACCTGACTTCAAACTATAC
```
